# Asymptomatic giant intrathyroid parathyroid carcinoma and common parathyroid carcinoma: two case reports and literature review

**DOI:** 10.3389/fsurg.2026.1793749

**Published:** 2026-08-06

**Authors:** Riqiang Liu, Weiwei Xie, Junzhang Hang, Mengyang Li

**Affiliations:** 1 Department of Thyroid Surgery, People’s Hospital of Guangxi Zhuang Autonomous Region, Nanning, Guangxi, China; 2 Department of Medical Oncology, People’s Hospital of Guangxi Zhuang Autonomous Region, Nanning, Guangxi, China

**Keywords:** endocrine surgery, hyperparathyroidism, intrathyroidal parathyroid carcinoma, parathyroid carcinoma, pathology

## Abstract

**Background:**

Parathyroid carcinoma (PC) is a rare, slow-growing but locally aggressive endocrine malignant tumor. Its clinical diagnosis is difficult, especially intrathyroid parathyroid carcinoma with atypical or asymptomatic symptoms. It is difficult to make a clear diagnosis before and during surgery.

**Case description:**

A 27-year-old female with elevated parathyroid hormone for 1 month showed a hypoechoic nodule immediately behind the middle of the left lobe of the thyroid gland. Laboratory work showed mild elevations in blood calcium and parathyroid hormone. She underwent parathyroid adenoma resection and thyroidectomy and functional central lymph node dissection. Her symptoms resolved after the operation and her recurrence rate remained negative at 20 months of follow-up. Another 74-year-old female patient with giant intrathyroid parathyroid carcinoma developed recurrence and metastasis only 5 months after undergoing radical thyroid cancer and total thyroidectomy.

**Conclusion:**

We compared the differences in clinical manifestations, imaging, pathological features, treatment and prognosis between two cases of parathyroid carcinoma without obvious clinical symptoms.

## Introduction

Parathyroid carcinoma (PC) is a rare endocrine tumor, accounting for less than 1% of sporadic primary hyperparathyroidism cases. It is mostly functional tumors that progress slowly but is locally aggressive. The main clinical manifestation is elevated PTH levels and associated hypercalcemia symptoms (such as bone disease, renal damage, arrhythmia, and neurocognitive disorders) (1, 2). Among them, non-functioning parathyroid carcinoma (PC) is very rare, accounting for only about 2% of all PC cases. It is characterized by normal serum calcium and PTH levels, and its clinical manifestations are dominated by local mass and invadement-related symptoms (3). Parathyroid surgery is the first choice for patients with PC. Early identification and complete resection of lesions are important to improve the prognosis of patients with PC and reduce mortality (4). This report compares a case of asymptomatic giant intrathyroid parathyroid carcinoma with a case of common parathyroid carcinoma, analyzes the differences between the two in clinical manifestations, imaging, pathological features, treatment and prognosis, and reviews the literature.

## Case details

### Case 1

**Figure 1:**
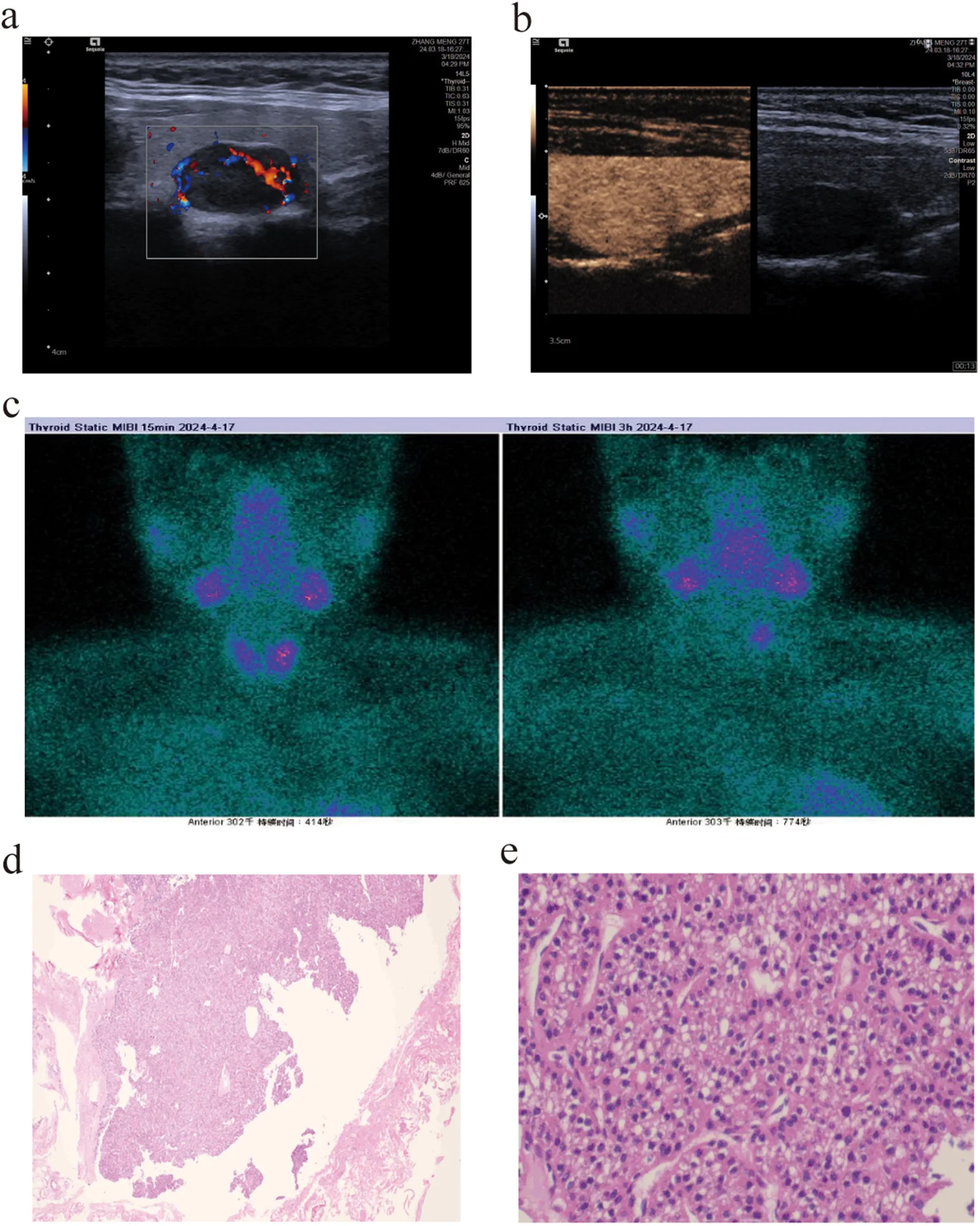
**(a,b)** Thyroid ultrasound. **(c)** ECT - ectopic parathyroid phenomenon. **(d,e)** Pathology.

The patient is a 27-year-old female who was admitted to the hospital in April 2024 due to elevated parathyroid hormone for more than one month. The patient's general condition was normal and there were no other symptoms. Physical examination: trachea was centered. No obvious mass was touched in the neck. Contrast-enhanced thyroid ultrasound: hypoechoic nodule closely behind the middle and rear of the left lobe of the thyroid gland, with narrow boundaries, and a range of approximately 17*12*11 mm (Figures 1a,b). Preoperative parathyroid stimulation (PTH) was 220.88pg/mL. Serum calcium: 2.87 mmol/L. ECT-Ectopic parathyroid phenomenon: Equivalent to a horizontal round-like abnormal tracer retention focus in the middle of the left lobe, considering hyperfunctional parathyroid tissue (Figure 1c).

After routine preoperative examination and preparation, endoscopic resection of parathyroid adenoma was performed under general anesthesia. During the operation, there was a mass about 15*14 mm in length and a complete capsule on the posterior and inferior part of the left thyroid gland. Intraoperative rapid freezing: parathyroid adenomatous tumor. The operation was successful and the patient recovered well after the operation. Post-operative laboratory tests: serum calcium: 2.26 mmol/L, serum phosphorus: 1.51 mmol/L, and PTH: 61.11 pg/mL, all within the normal range.

Paraffin pathology: Parathyroid carcinoma, the largest diameter of the tumor is 2.1 cm, partially breaks through the fibrous capsule to the surrounding fat tissue, and tumor thrombus in the vessel can be seen without nerve invasion. Please closely combine it with the clinical practice (Figures 1d,e). Immunohistochemistry: Tumor cells GATA-3(+), CgA(+), Bcl-2(+), CyclinD1 (-), E-cad (+), P53(about 1% weak +), Ki-67(about 2%+); D240(-), CD31 (+, vascular tumor thrombus can be seen). After pathological report, left thyroidectomy and functional dissection of central lymph nodes were added. Post-operative pathology showed that there was no cancer involvement in the thyroid tissue and no cancer metastasis in the central lymph nodes.

### Case 2

**Figure 2:**
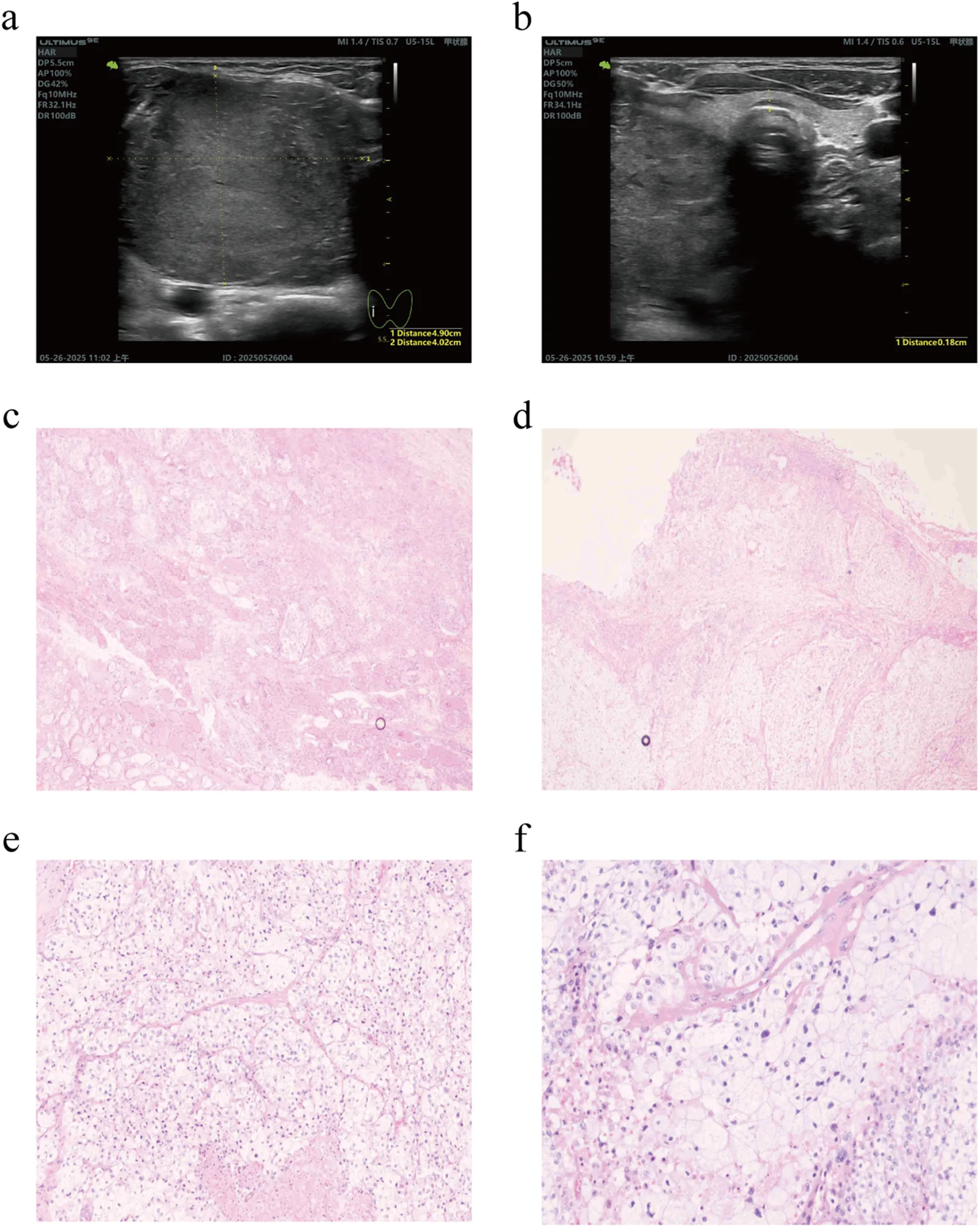
**(a,b)** Thyroid ultrasound. **(c,d)** Fast pathologic. **(e,f)** Pathology.

The patient is a 74-year-old female. She was admitted to the hospital in May 2025 due to a 2-year-old mass in the right neck. The patient had no dyspnea and no other symptoms. Physical examination: The trachea is left, and a painless mass of about 50mm*30 mm in size can be palpable in the right thyroid gland. The surface is smooth and the texture is tough, without obvious tenderness or tenderness, and the range of motion is moderate. It moves up and down with swallowing. Thyroid ultrasound: A cystic solid mass was seen in the right lobe of the thyroid gland with clear boundaries and uneven internal echoes. The size was about 59*49*40 mm, occupying almost the entire gland (Figures 2a,b). No special abnormalities were found in preoperative laboratory examinations.

After routine preoperative examination and preparation, right thyroidectomy was performed under general anesthesia. During the operation, a solid hard nodule about 6 cm in size was seen occupying the entire right thyroid gland and adhering tightly to surrounding tissues. Intraoperative rapid frozen section: malignant tumor, considered to be poorly differentiated cancer (Figures 2c,d). Subsequently, it was decided to perform radical resection of right thyroid cancer and total left thyroidectomy based on the results of intraoperative rapid frozen section. The operation was successful and the patient recovered well after the operation. Post-operative laboratory examination: serum phosphorus: 2.02 mmol/L, with no significant abnormalities.

Paraffin pathology: left thyroid tissue, approximately 65 × 33 × 6 mm in size. Right parathyroid carcinoma, with a maximum diameter of 6 cm, involved extracapsular fibrofatty tissue and thyroid tissue with vascular tumor thrombus, and no clear nerve invasion (Figures 2e,f). Immunohistochemistry: tumor cells GATA-3(+), CK(+), Syn(+), CD56(-), CgA (a few cells near the nucleus +),SF-1(a few+), MC(-), BRAF(-), CD163(-), Calcitonin(-), CEA[M](-), TPO(-), PAX-8(a small amount of weak+), TG(-), TTF-1(SPT24)(-), HMB45(-), Ki-67(about 70%+). No cancer metastasis was found in the right central lymph node.

## Discussion

Parathyroid adenocarcinoma is a rare disease with a low overall incidence, but recent studies have shown that the incidence of parathyroid glands in China has increased in recent years, reaching more than 5.0% (5, 6). In clinical practice, the diagnosis of parathyroid carcinoma is challenging. There are no reliable diagnostic criteria for parathyroid carcinoma before surgery. The final diagnosis requires the help of paraffin pathology and immunohistochemistry (7). In this report, the first patient was considered as a parathyroid adenoma before surgery, and the second patient was considered as a thyroid tumor before surgery. Both patients were diagnosed by pathology after surgery.

The cause of parathyroid cancer is currently unclear. Studies have shown that a rare autosomal dominant inherited disease-hyperparathyroidism-mandibular tumor syndrome (HPT-JT) may be related to the disease. As many as 15% of patients with this syndrome develop parathyroid cancer (8). It has been reported that mutations in the tumor suppressor gene CDC73 and abnormalities in the PI3 K/AKT/mTOR pathway are associated with the development of pancreatic cancer (PC). In addition, other recurrent genetic aberrations have been found in PC, including CCND1 overexpression, loss of heterozygosity or downregulation of genes such as glycogen synthase kinase 3β, retinoblastoma, adenomatous colonic polyposis, and tumor protein 53, and amplification of the EZH2 gene (8).

The main clinical manifestation of parathyroid carcinoma is elevated PTH levels and related hypercalcemia symptoms. In Case 1, the patient only presented with mild clinical symptoms before surgery, accompanied by mild increase in parathyroid hormone (PTH) and serum calcium levels. This is different from the previously reported typical biochemical characteristics of parathyroid carcinoma-namely, significant increase in PTH (often more than 3 to 10 times higher than the upper limit of normal) accompanied by severe hypercalcemia crisis (serum calcium > 3.50 mmol/L) (9). A study based on data analysis of 733 patients between 1985 and 2006 pointed out that the mortality rate of patients with parathyroid cancer was mainly related to hypercalcemia rather than tumor burden. Reports showed that the 5-year and 10-year survival rates of patients were 82.3% and 66% respectively (10). Case 1 was followed up for 20 months postoperatively, and his general condition was good without recurrence or metastasis.

However, in case 2, parathyroid hormone and serum calcium were within the normal range before surgery, but metastasis to both lungs occurred only 5 months after surgery. The lesion involved the right cricoid cartilage, internal jugular vein, upper trachea, and significantly compressed trachea. Partial metastasis of the right cervical lymph nodes were considered. 99mTc-MDP bone imaging showed slightly active neck metabolism, which was considered due to primary tumor infiltration. Clinically, non-functional parathyroid carcinoma (PC) is rare, and its symptoms are mostly related to local tumor infiltration and compression. Common manifestations include neck mass, hoarseness, and difficulty swallowing (3). Early diseases often have no typical symptoms, a characteristic that often leads to delayed diagnosis and exacerbates disease progression. Previous studies have shown that metastatic spread often occurs late in the course of the disease, involving cervical lymph nodes, lungs, liver and other sites (11). The neuroendocrine markers CgA and Syn are often expressed in parathyroid tumors. When the Ki-67 index is greater than 5%, it is necessary to be vigilant for the possibility of malignancy (12). However, there have been reports that a high KI-67 index may be an indicator of poor prognosis, but more data is needed to further confirm it. Postoperative pathology of patient 2 showed that the Ki-67 index was as high as about 70%, and vascular tumor thrombus was visible; while the Ki-67 index of patient 1 with a good prognosis was only about 2%. The comparison between the two indirectly revealed the reasons for the poor prognosis of patient in Case 2.

In summary, parathyroid adenocarcinoma is a rare malignant tumor. Due to the difficulty of diagnosis before and even during surgery, especially intrathyroid parathyroid carcinoma with insignificant clinical symptoms or asymptomatic, both are rare and challenging diagnoses. Surgery is still the first choice of treatment, and long-term close follow-up must be performed after surgery to detect recurrence or metastasis as soon as possible.

## Data Availability

The original contributions presented in the study are included in the article/Supplementary Material, further inquiries can be directed to the corresponding author.

## References

[1] Salcuni AS Cetani F Guarnieri V Nicastro V Romagnoli E De Martino D. Parathyroid carcinoma. Best Pract Res Clin Endocrinol Metab. (2018) 32(6):877–889. 10.1016/j.beem.2018.11.002 30551989

[2] Cetani F Pardi E Marcocci C. Update on parathyroid carcinoma. J Endocrinol Investig. (2016) 39(6):595–606. 10.1007/s40618-016-0447-3 27001435

[3] Cetani F Frustaci G Torregrossa L Magno S Basolo F Campomori A. A nonfunctioning parathyroid carcinoma misdiagnosed as a follicular thyroid nodule. World J Surg Oncol. (2015) 13:270. 10.1186/s12957-015-0672-9 26350418 PMC4563849

[4] Xue S Chen H Lv C Shen X Ding J Liu J. Preoperative diagnosis and prognosis in 40 parathyroid carcinoma patients. Clin Endocrinol (Oxf). (2016) 85(1):29–36. 10.1111/cen.13055 26939543

[5] Zhao L Liu J- He X-Y Zhao H- Sun L- Tao B. The changing clinical patterns of primary hyperparathyroidism in Chinese patients: data from 2000 to 2010 in a single clinical center. J Clin Endocrinol Metab. (2013) 98(2):721–728. 10.1210/jc.2012-2914 23365127

[6] Miao X. Parathyroid carcinoma: report of 10 patients and literature review. Neuro Endocrinol Lett. (2022) 43(4):233–238. 36528886

[7] Cetani F Pardi E Marcocci C. Parathyroid carcinoma. Front Horm Res. (2019) 51:63–76. 10.1159/000491039 30641523

[8] Cetani F Pardi E Marcocci C. Parathyroid carcinoma: a clinical and genetic perspective. Minerva Endocrinol. (2018) 43(2):144–155. 10.23736/S0391-1977.17.02737-7 28949121

[9] Lazzaro A Zhao GQ Kulke M. Diagnosis and management of parathyroid carcinoma. Clin Pharmacol Ther. (2024) 116(6):1496–1505. 10.1002/cpt.3432 39234888

[10] Asare EA Sturgeon C Winchester DJ Liu L Palis B Perrier ND. Parathyroid carcinoma: an update on treatment outcomes and prognostic factors from the national cancer data base (NCDB). Ann Surg Oncol. (2015) 22(12):3990–3995. 10.1245/s10434-015-4672-3 26077914

[11] Kubal M Lech M Lajeunesse-Trempe F Drakou EE Grossman AB Dimitriadis GK. Advances in the management of parathyroid carcinoma. Mol Cell Endocrinol. (2024) 592:112329. 10.1016/j.mce.2024.112329 38996836

[12] Erickson LA Mete O. Immunohistochemistry in diagnostic parathyroid pathology. Endocr Pathol. (2018) 29(2):113–129. 10.1007/s12022-018-9527-6 29626276

